# Impact of hypoglycemic regimens on cardiac recovery and quality of life after PCI in elderly AMI patients

**DOI:** 10.3389/fcvm.2026.1644294

**Published:** 2026-04-30

**Authors:** Lei Qian, Ping Zhou, Zhimin Zhao

**Affiliations:** 1 Department of Cardiology, The Affiliated Cardiovascular Hospital of Qingdao University, Qingdao, Shandong, China; 2 Trauma and Neurosurgery Department, Women and Children’s Hospital, Qingdao University, Qingdao, Shandong, China; 3 Department of Cardiology, Hongsen Hospital, Harbin Medical University, Sanya, Hainan, China

**Keywords:** acute myocardial infarction, cardiac function recovery, glycemic control, percutaneous coronary intervention, prognosis

## Abstract

**Objective:**

To investigate the impact of blood glucose control levels on cardiac function recovery and long-term prognosis in elderly patients with acute myocardial infarction (AMI) undergoing percutaneous coronary intervention (PCI).

**Method:**

A retrospective study included 80 elderly AMI patients from January 2023 to January 2024, divided into good (HbA1c ≤ 7%, *n* = 39) and poor (HbA1c > 7%, *n* = 41) baseline glucose control groups. Further, based on HbA1c at 3 months post-PCI, patients were classified into four groups: continuous good control (*n* = 34), postoperative improvement (*n* = 15), postoperative deterioration (*n* = 5), and persistent poor control (*n* = 26). Outcomes compared included MACE, stent thrombosis, cardiovascular readmission, NYHA class improvement, eGFR decline >20%, LVEF, and quality of life scores. Patients were prospectively followed at 3, 6, and 12 months after PCI via standardized telephone interviews or in-person outpatient visits to assess glycemic control (HbA1c, fasting glucose), cardiac function (LVEF, NYHA class), MACE, renal function, and quality of life (SF-36).

**Result:**

The poor baseline control group had higher diabetes prevalence (78.05% vs. 20.51%, *P* < 0.001) and more frequent use of metformin and insulin. Group 4 had the highest MACE incidence (34.62%), lowest mean LVEF (45.3 ± 6.1%), worst renal function preservation, and lowest quality of life. Group 3 showed increased MACE risk (20%). In contrast, Group 2, achieving glucose control postoperatively, had a prognosis similar to Group 1.

**Conclusion:**

Continuous blood glucose management after PCI significantly improves prognosis in elderly AMI patients. Dynamic monitoring provides greater clinical value than baseline control alone.

## 1 Introduction

Acute Myocardial Infarction (AMI) is one of the main causes of death and disability worldwide (1). With the intensification of population aging, the incidence of AMI in the elderly has been increasing year by year. According to statistics, elderly AMI patients in China account for more than 60% of all cases, and the in-hospital mortality rate is as high as 10% to 15% (2). Percutaneous coronary intervention (PCI), as an important means of revascularization, can effectively reduce the mortality rate in the acute phase. However, problems such as poor postoperative cardiac function recovery and high incidence of major adverse cardiovascular events (MACE) remain prominent, seriously affecting the long-term quality of life and prognosis of patients (3). It is notable that approximately 40% of elderly AMI patients are complicated with diabetes. The hyperglycemic state aggravates myocardial ischemia-reperfusion injury through mechanisms such as pro-inflammatory, pro-oxidative stress and endothelial dysfunction, resulting in accelerated left ventricular remodeling, microcirculation disorders and an increased risk of in-stent restenosis (4, 5). However, at present, clinical attention to blood glucose management in elderly AMI patients is mostly focused on the control in the acute phase, while the impact of long-term dynamic changes in blood glucose after PCI on cardiac function recovery and prognosis remains unclear.

In recent years, the cardiovascular protective effect of hypoglycemic drugs has attracted much attention. Clinical evidence shows (6, 7) that there is significant heterogeneity in the effects of different hypoglycemic regimens on cardiovascular outcomes: SGLT-2 inhibitors (such as empagliflozin) have been proven to reduce the risk of hospitalization for heart failure; GLP-1 receptor agonists (such as liraglutide) can reduce the occurrence of MACE; However, intensive insulin therapy may increase the risk of hypoglycemia and instead lead to a deterioration of prognosis. Most of the existing studies have focused on patients with chronic heart failure or stable coronary heart disease. There is still a lack of evidence-based basis for the application timing of PCI treatment in elderly AMI patients, the differences in efficacy compared with traditional hypoglycemic regimens (such as insulin and sulfonylureas), and the predictive value for cardiac function recovery. In addition, elderly patients often have problems such as multimedication, reduced liver and kidney function, and susceptibility to hypoglycemia. How to formulate individualized hypoglycemic strategies to balance blood sugar control and safety has become a key challenge in improving prognosis. Most of the existing studies focus on the association between baseline blood glucose levels and postoperative outcomes after PCI, but ignore the differentiated effects of the dynamic evolution of postoperative blood glucose control on cardiac function and quality of life. More importantly, different hypoglycemic medication regimens may independently affect the recovery of cardiac function through pathways such as regulating myocardial energy metabolism and inhibiting fibrosis. The specificity of this mechanism in the elderly population has not been fully clarified. Therefore, this study aims to reveal the predictive effect on postoperative cardiac function recovery and quality of life in elderly AMI patients by analyzing the dynamic changes of blood glucose control and the selection of hypoglycemic drugs before and after PCI treatment, and to provide a theoretical basis for optimizing the integrated management of cardiovascular metabolism in the elderly.

## 2 Methods

### 2.1 Study the flowchart

**Figure 1:**
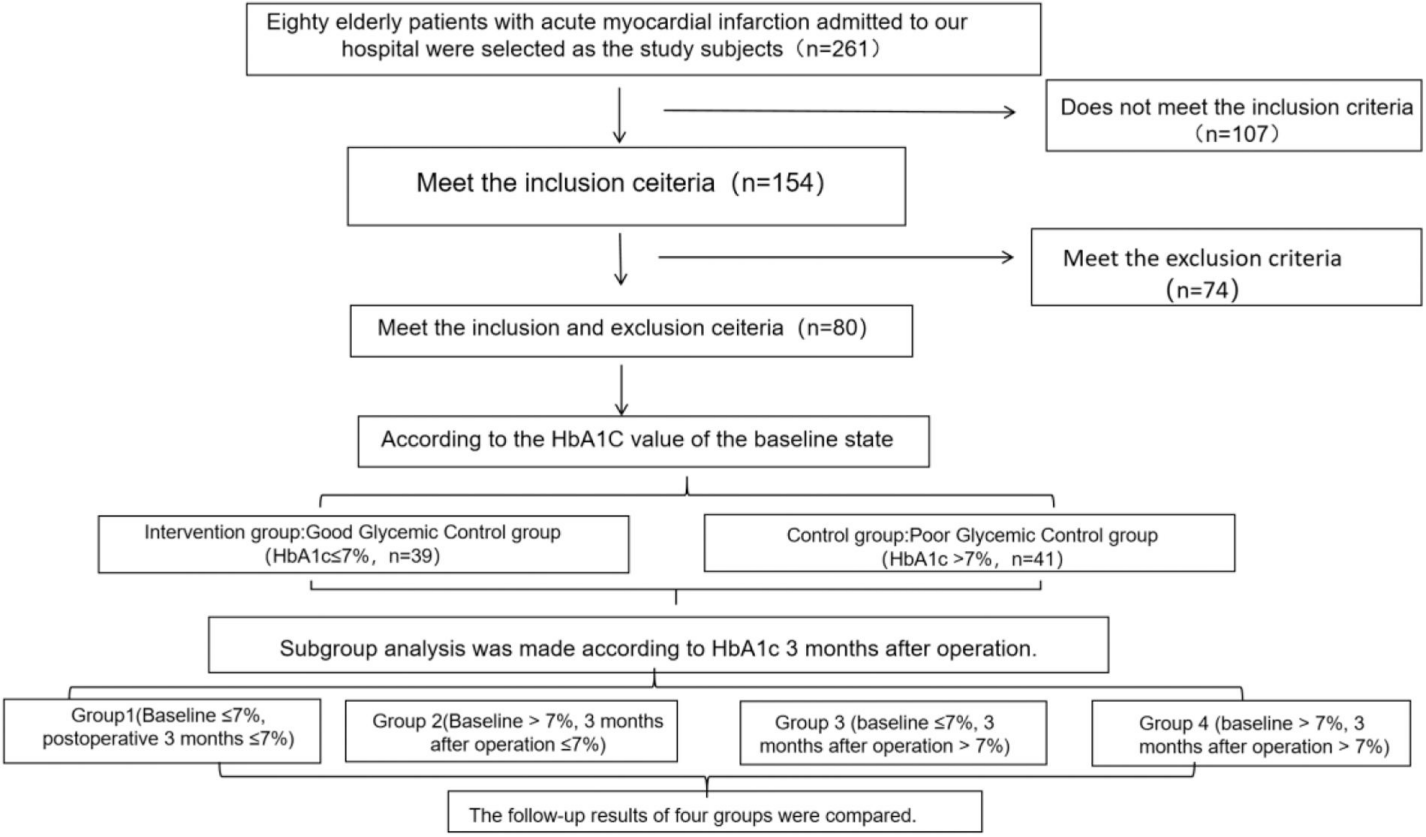
Research flowchart.

Figure 1 shows the flow chart of this research.

### 2.2 Clinical data

Eighty elderly patients with acute myocardial infarction admitted to our hospital from January 2023 to January 2024 were selected as the research subjects. Firstly, they were divided into the group with good baseline blood glucose control (HbA1c ≤ 7%, *n* = 39) and the group with poor blood glucose control (HbA1c > 7%, *n* = 41) based on the HbA1c values at baseline. The general data of the two groups of patients are detailed in Table 1. There was no statistical significance in the comparison of clinical characteristics such as gender, age, BMI, smoking history, drinking history, and hypertension (*P* > 0.05). The current study was approved by the Ethics Committee of the Hongsen Hospital, Harbin Medical University. Written informed consents from all patients were obtained in any experimental work with humans.

**Table 1:** Baseline characteristics comparison ($\bar{x}\pm s$, n/%).

Project	The group with good blood sugar control (*n* = 39)	Poor blood sugar control group (*n* = 41)	*T* value/*χ^2^* value	*P* value
Basic demographic information				
Gender (Male/Female)	22 (56.41)	24 (58.54)	0.037	0.848
Age (years,$\bar{x}\pm s$)	68.51 ± 6.34	70.22 ± 6.95	1.148	0.255
BMI (kg/m^2^, $\bar{x}\pm s$))	24.95 ± 2.93	26.08 ± 3.41	1.586	0.117
History of smoking (Yes, %)	15 (38.46)	20 (48.78)	0.865	0.352
Hypertension (yes, %)	28 (71.79)	34 (82.93)	1.420	0.233
Diabetes (Yes, %)	8 (20.51)	32 (78.05)	26.467	<0.001
Fasting blood glucose (mmol/L, $\bar{x}\pm s$))	6.42 ± 0.56	6.81 ± 1.15	1.913	0.060
HbA1c (%,$\bar{x}\pm s$))	6.94 ± 0.55	7.49 ± 1.02	2.980	0.004
Total cholesterol (mmol/L, $\bar{x}\pm s$))	4.82 ± 1.07	5.06 ± 1.13	0.974	0.333
LDL-C (mmol/L, $\bar{x}\pm s$))	2.55 ± 0.82	2.83 ± 1.01	1.357	0.179
HDL-C (mmol/L, $\bar{x}\pm s$))	1.33 ± 0.35	1.23 ± 0.32	1.335	0.186
Triglyceride (mmol/L, $\bar{x}\pm s$))	1.65 ± 0.92	1.91 ± 1.14	1.119	0.267
Blood creatinine (*μ*mol/L, $\bar{x}\pm s$))	77.85 ± 14.42	84.33 ± 17.65	1.793	0.077
eGRR (mL/min/1.73m^2^, $\bar{x}\pm s$))	76.43 ± 13.18	72.85 ± 15.77	1.099	0.275
LVEF (%, $\bar{x}\pm s$))	54.63 ± 7.35	51.42 ± 8.05	1.860	0.067
Killip grade ≥2 (Yes, %)	8 (20.51)	16 (39.02)	3.262	0.071
Reinfarction during hospitalization (Yes, %)	2 (5.13)	5 (12.20)	1.250	0.264
Cardiogenic shock during hospitalization (Yes, %)	2 (5.13)	7 (17.07)	2.856	0.091
Restenosis after PCI (Yes, %)	4 (10.26)	9 (21.95)	2.009	0.156
Stent thrombosis after PCI (Yes, %)	1 (2.56)	3 (7.32)	0.951	0.330
History of tumor (Yes, %)	2 (5.13)	3 (7.32)	0.163	0.686

Inclusion criteria: (1) Elderly patients diagnosed with acute myocardial infarction (AMI) (8); (2) Age ≥65 years old, gender not limited; (3) Percutaneous coronary intervention (PCI) was successfully completed after the onset of the disease; (4) The baseline HbA1c data was complete, and the patients or their families signed the informed consent form, voluntarily participated in the study and completed the follow-up.

Exclusion criteria: (1) Those with severe liver and kidney dysfunction; (2) Those with active malignant tumors or other serious non-cardiovascular diseases; (3) The expected survival period is less than one year, or there is a risk of loss to follow-up; (4) Participate in other clinical trials that may interfere with this study at that time; (4) There are contraindications for PCI surgery (such as severe coagulation dysfunction) or the target blood vessels were not successfully opened during the operation; (5) Accompanied by cognitive impairment or mental illness.

### 2.3 Data collection

The baseline data of the included subjects were collected using the electronic medical record system in the hospital. Including demographic information [gender, age, body mass index (BMI), smoking history, drinking history, comorbidities (hypertension, diabetes, tumor history, etc.)], clinical indicators [metabolic indicators (fasting blood glucose, HbA1c, total cholesterol, LDL-C, HDL-C and triglycerides), cardiac and renal functions (left ventricular ejection fraction (LVEF), Killi p classification, serum creatinine, estimated glomerular filtration rate (eGFR), and therapeutic characteristics [stent type during PCI, postoperative medication (antiplatelet drugs, statins, beta-blockers, hypoglycemic drugs, etc.)]. The patients were followed at 3, 6, and 12 months after the procedure. Follow-up was conducted via standardized telephone interviews or in-person outpatient visits. Assessments included glycemic control (HbA1c, fasting glucose), cardiac function (LVEF, NYHA class) (9), MACE (reinfarction, restenosis, cardiogenic death), quality of life (SF-36) (10) and renal function (eGFR; >20% decline defined deterioration).

#### 2.3.1 Medication exposure and adherence ascertainment

For each antihyperglycemic class (metformin, sulfonylureas, DPP-4 inhibitors, SGLT-2 inhibitors, GLP-1 receptor agonists, insulin), we captured start/stop dates, doses, and up-/down-titrations at baseline and at 3, 6, and 12 months. Adherence was assessed at each visit by a brief structured interview (self-reported proportion of doses taken during the prior 7 days and presence of any ≥2 consecutively missed doses). Patients reporting ≥80% doses taken were categorized as “adherent”; others were labeled “suboptimal adherence.” These variables were used descriptively and in exploratory adjusted analyses.

### 2.4 Standardization of intervention and treatment

All patients received coronary angiography and PCI treatment. During the operation, drug-eluting stents (DES) were selected based on the vascular lesion conditions. After the operation, dual antiplatelet therapy (aspirin + clopidogrel/ticagrelor), statins and beta-blockers were used as recommended by the guidelines. None of the patients were forcibly intervened in the hypoglycemic regimen. However, the actual medication used by the patients (such as metformin, insulin, SGLT-2 inhibitors, etc.) was recorded, and the treatment was adjusted according to clinical needs.

#### 2.4.1 Operational criteria for glucose management during follow-up

This was an observational study; treating teams were not protocol-mandated to use any specific antihyperglycemic regimen. To improve reproducibility of our comparisons, we prespecified operational definitions used to classify real-world management changes at each follow-up visit (3, 6, and 12 months). “Intensification” was defined as (i) initiation of any glucose-lowering agent not used at baseline or (ii) up-titration of dose in response to HbA1c > 7.0% or fasting plasma glucose (FPG) > 7.0 mmol/L confirmed on two measurements, aligned with contemporary diabetes guidance. “De-intensification” referred to dose reduction or drug discontinuation due to HbA1c ≤ 6.5%, recurrent hypoglycemia, frailty, or renal decline. Initiation of SGLT-2 inhibitors was favored when estimated glomerular filtration rate (eGFR) ≥ 45 mL/min/1.73 m^2^, while basal insulin was considered for symptomatic hyperglycemia or HbA1c ≥ 9% despite oral therapy. Adherence support (education, simplified regimens) was routinely offered.

Follow-up assessments were conducted via standardized telephone or in-person visits, during which current medications, dose changes since the prior visit, and reasons for changes were recorded using structured case-report forms.

### 2.5 Statistical analysis

SPSS 21.0 statistical software was applied. Before statistical analysis, the measurement data were all subjected to normal distribution and homogeneity of variance analysis to test if they met the requirements of normal distribution or approximately normal distribution, expressed as $\bar{x}\pm s$. The t-test was used for comparison between the two groups, and one-way analysis of variance (ANOVA) or Kruskal–Wallis test was used for comparison among the four groups. Pairwise comparisons were made using Bonferroni correction; to address multiplicity across several outcomes compared in four groups, we controlled the family-wise error rate by applying Bonferroni adjustment to all *post-hoc* pairwise tests for both continuous and categorical endpoints. We prespecified MACE and LVEF as primary endpoints; other outcomes were considered secondary and exploratory, with two-sided *α* maintained at 0.05. Take example n (%) to represent the count data and conduct the *χ*2 test; rank sum test was used for grade data, and repeated measures analysis of variance was used for the comparison of repeated measurement data. A *P* value <0.05 indicated a statistically significant difference.

#### 2.5.1 Adjustment for potential confounders

In exploratory multivariable models, we evaluated whether the association between 3-month glycemic status groups and outcomes persisted after adjustment for age, sex, body mass index, smoking, hypertension, diabetes status, baseline LVEF, Killip class, baseline eGFR, and use of statins and β-blockers. Binary outcomes (e.g., MACE) were modeled with logistic regression; continuous outcomes (e.g., LVEF, SF-36) with generalized linear models. These analyses were prespecified as supportive and did not replace the primary comparisons; conclusions were qualitatively unchanged.

## 3 Results

### 3.1 Baseline period data

The prevalence of diabetes in the poor blood glucose control group was significantly higher than that in the good control group (78.05% vs. 20.51%, *P* < 0.001), and its baseline HbA1c and fasting blood glucose levels were higher, suggesting that the diabetic status was the core factor for the difference in blood glucose control. In addition, there were no significant differences between the two groups in key indicators such as lipid profile, renal function (eGFR), and left ventricular ejection fraction (LVEF) (*P* > 0.05). However, in the poorly controlled group, the Killip grade ≥2 and the incidence of cardiogenic shock during hospitalization showed an increasing trend, suggesting that abnormal baseline blood glucose may be associated with more severe myocardial injury. See Table 1.

### 3.2 Comparison of the usage patterns of hypoglycemic drugs in the two groups

The characteristics of intensive hypoglycemic treatment in the poor blood glucose control group were significant. The usage rates of metformin (53.66% vs. 25.64%, *P* = 0.011) and insulin (29.27% vs. 10.26%, *P* = 0.034) in the group were significantly higher than those in the well-controlled group. However, the application proportion of new drugs such as SGLT-2 inhibitors was generally low (9.76% vs. 2.56%). It is indicated that the disease course of diabetes in the poorly controlled group is longer or the islet function is worse. However, it is also indicated that the effect of traditional hypoglycemic regimens (such as insulin) on achieving blood glucose targets is limited. See Table 2.

**Table 2:** Comparison of therapeutic medication in the two groups.

Medication category	The group with good blood sugar control(*n* = 39)	Poor blood sugar control group(*n* = 41)	*χ^2^* value	*P* value
Heart-related medications (%)				
Aspirin (%)	32 (82.05)	34 (82.93)	0.011	0.918
Clopidogrel/ticagrelor (%)	29 (74.36)	31 (75.61)	0.017	0.897
Statins (atorvastatin/rosuvastatin) (%)	37 (94.87)	38 (92.68)	0.163	0.686
Beta-blockers (metoprolol/bisoprolol) (%)	28 (71.79)	30 (73.17)	0.019	0.890
ACEI/ARB (Benazepril/Irbesartan, etc.)(%)	26 (66.67)	31 (75.61)	0.780	0.377
Calcium channel antagonists (amlodipine/nifedipine) (%)	12 (30.77)	18 (43.90)	1.471	0.225
Diuretics (furosemide/spironolactone) (%)	8 (20.51)	14 (31.71)	1.863	0.172
Nitrates (isosorbide mononitrate/nitroglycerin (%)	9 (23.08)	13 (53.66)	0.747	0.388
Hypoglycemic drugs				
Metformin (%)	10 (25.64)	22 (53.66)	6.538	0.011
Sulfonylureas (Gliclazide/glimepiride) (%)	4 (10.26)	9 (21.95)	2.009	0.156
DPP-4 inhibitors (sitagliptin/vigagliptin) (%)	2 (5.13)	6 (14.63)	2.007	0.157
SGLT-2 inhibitors (dapagliflozin/empagliflozin) (%)	1 (2.56)	4 (9.76)	1.764	0.184
Insulin therapy (%)	4 (10.26)	12(29.27)	4.515	0.034

### 3.3 Blood glucose control three months after PCI treatment

**Figure 2:**
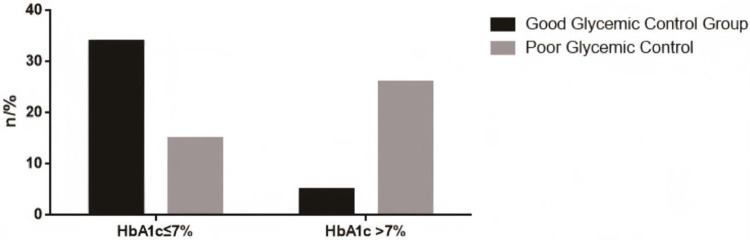
Blood glucose control 3 months after PCI treatment.

In the group with good baseline blood glucose control (HbA1c ≤ 7%), 87.18% (34/39) of the patients maintained good control, while 12.82% (5/39) deteriorated (HbA1c > 7%). In the group with poor baseline control (HbA1c > 7%), 36.59% (15/41) of the patients achieved blood glucose HbA1c ≤ 7% through postoperative intervention, but 63.41% (26/41) still had poor control. See Figure 2.

### 3.4 Comparison of clinical outcomes

**Figure 3:**
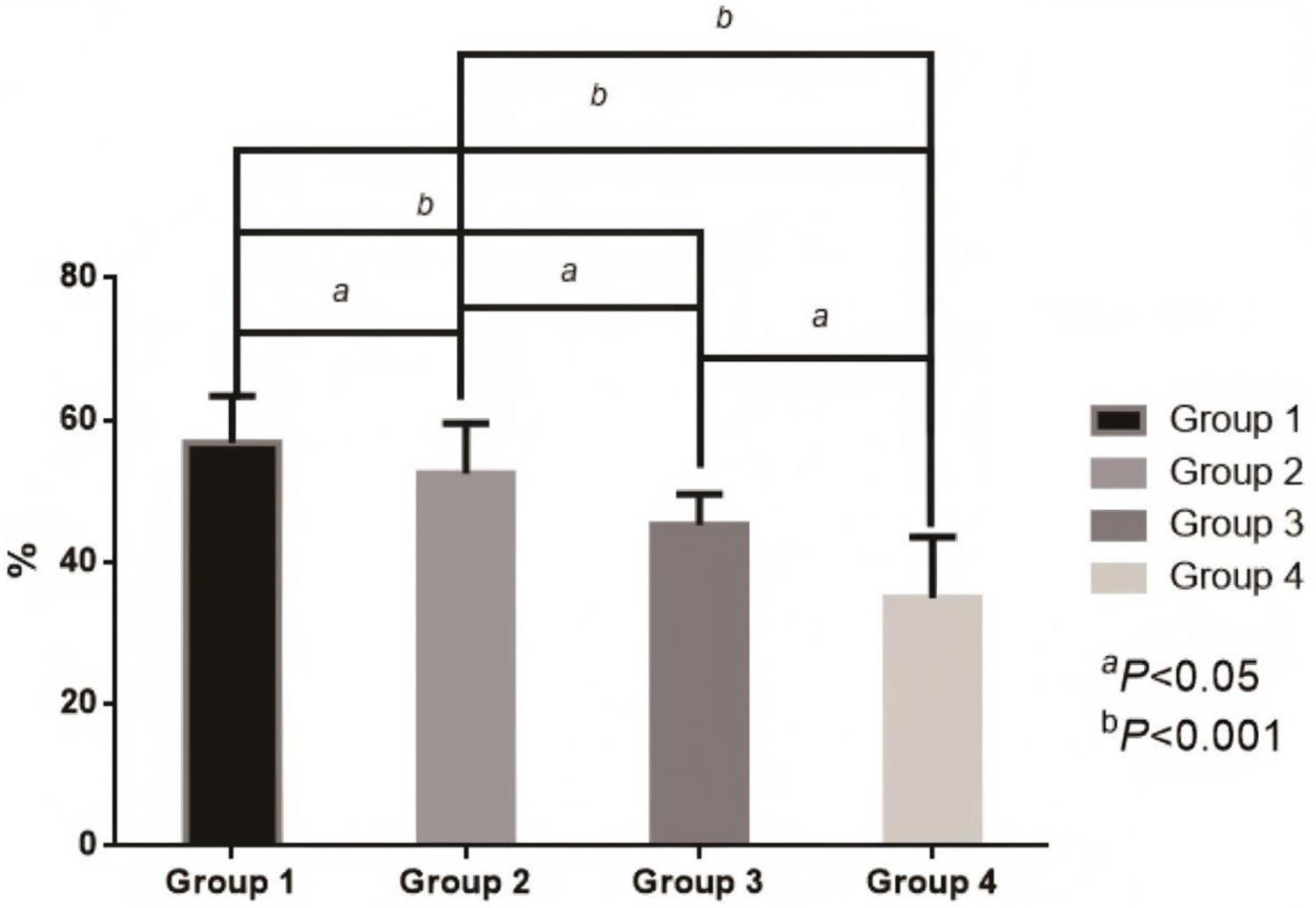
Comparison of four groups of LVEF values. Group 1: Good continuous blood glucose control (baseline ≤7%, ≤7% at 3 months after surgery); Group 2: Postoperative improvement group (baseline >7%, ≤7% at 3 months after surgery); Group 3: Postoperative deterioration group (baseline ≤7%, >7% at 3 months after surgery); Group 4: Persistent poor blood glucose control (baseline >7%, >7% at 3 months after surgery).

**Figure 4:**
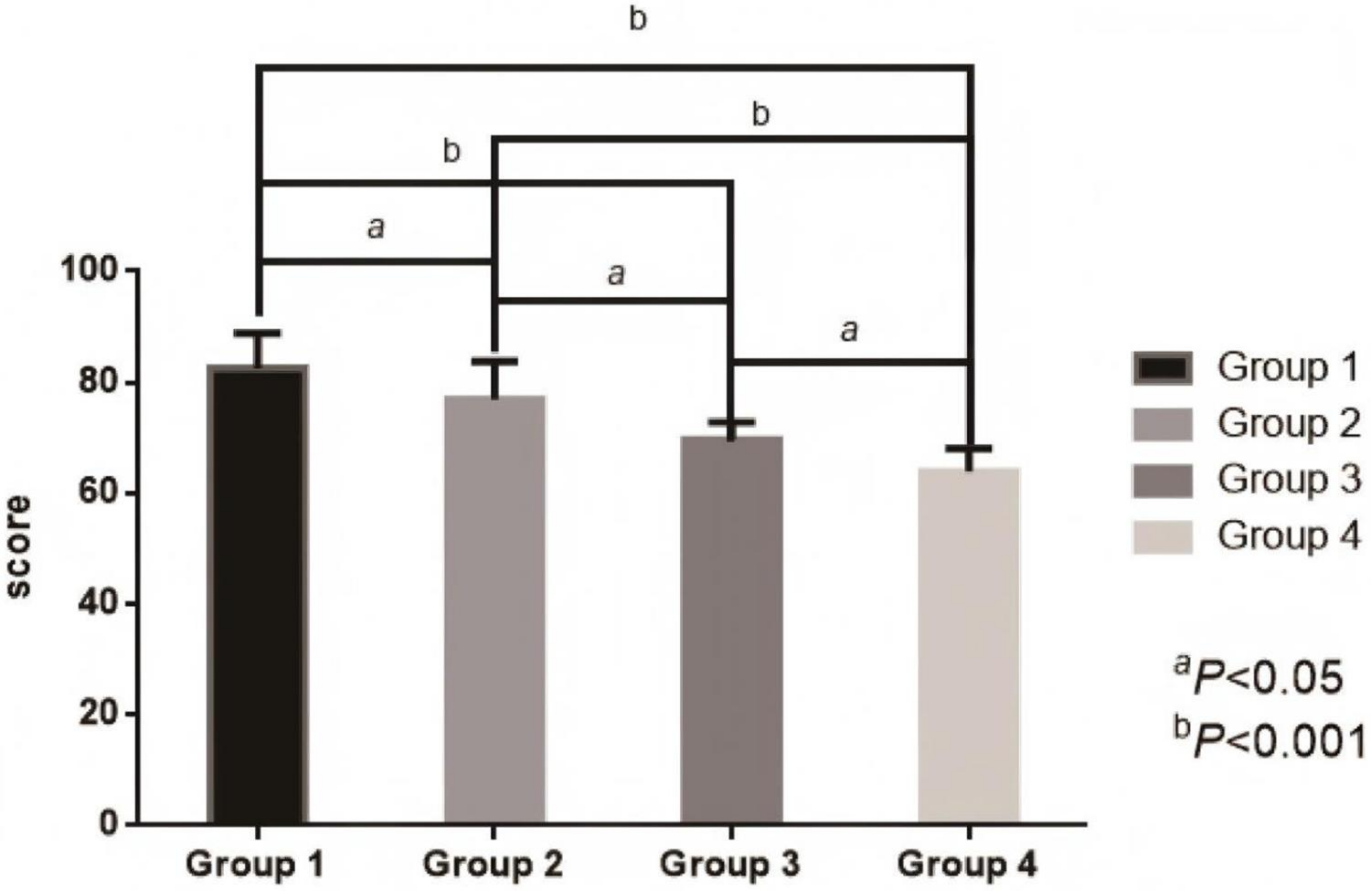
Comparison of SF-36 scores among the four groups.

After dynamic grouping, the influence of blood glucose control status 3 months after surgery on the prognosis at 1 year showed a gradient effect: The incidence of MACE (53.85%, 14/26), restenosis rate (23.08%, 6/26), and readmission rate due to cardiovascular events (30.77%, 8/26) in the persistently poorly controlled group (Group 4) were significantly higher than those in other groups. Moreover, its mean LVEF and SF-36 score were the lowest, and the improvement rate of cardiac function (38.46%). 10/26) and renal function protection [eGFR decline >20% accounted for 26.92% (7/26)] were also the poorest. It is notable that the outcome after the operation (Group 2) was close to that of the group with good continuous control (Group 1), and its MACE risk, LVEF and quality of life score were significantly better than those of group 4, confirming that active blood glucose control after the operation can partially reverse the adverse effects of baseline blood glucose. It is suggested that HbA1c 3 months after PCI is a key indicator for predicting cardiovascular prognosis in 1 year, and is even more important than baseline HbA1c. Furthermore, the risks of MACE (60%, 3/5), cardiogenic death (20%, 1/5), and non-fatal myocardial infarction (20%, 1/5) in the group with good baseline control but postoperative deterioration (Group 3) were still significantly increased, suggesting that postoperative blood glucose fluctuations may offset the benefits of early treatment. See Table 3 and Figures 3, 4.

**Table 3:** Comparison of clinical outcomes.

Indicator	Group 1 (*n* = 34)	Group 2 (*n* = 15)	Group 3 (*n* = 5)	Group 4 (*n* = 26)	*χ^2^*-value	*P*-value
Major cardiovascular events (MACE, %)	4 (11.76)	3 (20.00)	3 (60.00)	14 (53.85)	15.218	0.002
Cardiogenic death (%)	1 (2.94)	1 (6.67)	1 (20.00)	3 (11.54)	2.771	0.428
Non-fatal myocardial infarction (%)	1 (2.94)	1 (6.67)	1 (20.00)	5 (19.23)	5.085	0.166
Restenosis after PCI (%)	2 (5.88)	1 (6.67)	1 (20.00)	6 (23.08)	4.745	0.191
Stent thrombosis (%)	1 (2.94)	1 (6.67)	1 (20.00)	3 (11.54)	2.771	0.428
Readmission due to cardiovascular events (%)	3 (8.82)	2 (13.33)	1 (20.00)	8 (30.77)	5.146	0.161
The NYHA cardiac function classification has improved (%)	26 (76.47)	11 (73.33)	3 (60.00)	10 (38.46)	12.243	0.007
eFGR has decreased by more than 20% (%)	2 (5.88)	1 (6.67)	1 (20.00)	7 (26.92)	6.378	0.095
The mean value of LVEF 12 months after the operation (%)	56.83 ± 6.54	52.51 ± 7.05	45.24 ± 4.33	34.94 ± 8.62	46.568	<0.001
SF-36 score (points)	82.54 ± 6.26	76.91 ± 6.84	69.55 ± 3.26	63.86 ± 4.15	55.977	<0.001

Group 1: Good continuous blood glucose control (baseline ≤7%, ≤7% at 3 months after surgery); Group 2: Postoperative improvement group (baseline >7%, ≤7% at 3 months after surgery); Group 3: Postoperative deterioration group (baseline ≤7%, >7% at 3 months after surgery); Group 4: Persistent poor blood glucose control (baseline >7%, >7% at 3 months after surgery).

## 4 Discussion

AMI remains one of the serious cardiovascular diseases that cause death and disability in elderly patients at present. On this basis, elderly AMI patients with diabetes have a higher risk of poor prognosis. The literature indicates (11, 12) that the hyperglycemic state can significantly aggravate myocardial ischemia-reperfusion injury through multiple pathological mechanisms such as activating pro-inflammatory responses, intensifying oxidative stress injury, and disrupting endothelial functional homeostasis, thereby leading to accelerated left ventricular remodeling, delayed cardiac function recovery, and an increased incidence of major adverse cardiovascular events (MACE). Although percutaneous coronary intervention (PCI), as a key approach for revascularization, has significantly improved the acute survival rate of AMI patients, long-term prognostic issues such as the progression of postoperative cardiac insufficiency and the decline in quality of life still need to be urgently addressed. Based on this, this study conducted a retrospective analysis of the clinical data of 80 elderly AMI patients, aiming to provide key evidence-based evidence for the optimization of individualized hypoglycemic treatment strategies for AMI patients. Our findings integrate with recent literature in cardiometabolic cardiology. In nationwide data, use of cardioprotective glucose-lowering drugs (SGLT-2 inhibitors, GLP-1 receptor agonists) among patients with diabetes and coronary artery disease has increased but remains suboptimal, aligning with our cohort's low uptake of SGLT-2 inhibitors (13, 14).

Moreover, diabetes relates heterogeneously to revascularization outcomes: a 2024 meta-analysis showed diabetes is associated with higher primary restenosis risk after endovascular treatment, while a 2025 left-main PCI registry found no overall mortality difference by diabetes status but signaled risk in specific subgroups—contextualizing our observation that persistent poor glycemic control, rather than diabetes *per se*, tracked worst outcomes (15, 16).

The results of this study show that patients in the poor blood glucose control group are more inclined to adopt traditional hypoglycemic regiments such as metformin (53.66%) and insulin (29.27%), while the application ratios of new drugs such as SGLT-2 inhibitors (9.76%) and GLP-1 receptor agonists are significantly lower. The analysis of the reason lies in that although traditional hypoglycemic drugs can rapidly regulate blood sugar, their hypoglycemic risk and potential pro-atherosclerotic effects may weaken the long-term cardiovascular benefits (17); Conversely, new drugs such as SGLT-2 inhibitors have been proven to reduce the risk of hospitalization for heart failure and improve cardiac function through mechanisms such as regulating myocardial energy metabolism and inhibiting fibrosis (18, 19). This phenomenon is consistent with the results of several randomized controlled studies in recent years, suggesting that optimizing drug selection is of key significance for improving the prognosis of elderly AMI patients. Dynamic follow-up data showed that the control status of HbA1c at 3 months after the operation was significantly gradient associated with the clinical outcome at 1 year. The incidence of MACE (53.85%), restenosis rate (23.08%), and readmission rate (30.77%) in the persistent poor blood glucose control group (Group 4) were significantly higher than those in other subgroups, and its LVEF value and SF-36 score were also the lowest in each group. This result is basically consistent with previous mechanism studies (20, 21), that is, persistent hyperglycemia accelerates the process of myocardial fibrosis and disrupts microcirculation homeostasis by activating the oxidative stress chain reaction, the release of pro-inflammatory factors and endothelial dysfunction, ultimately leading to the deterioration of left ventricular remodeling and an increased risk of cardiovascular events. The results of this study further emphasize the core role of postoperative blood glucose stability management in blocking the pathological pathways of myocardial injury.

Our gradient association between 3-month HbA1c status and 1-year outcomes aligns with reports that persistent hyperglycemia worsens ventricular remodeling and MACE risk in AMI populations (11, 12, 20, 21). Consistent with observational data and meta-analyses, the low uptake yet potential benefit of SGLT-2 inhibitors observed in our cohort echoes prior evidence showing reduced heart-failure hospitalization and improved cardiac function with SGLT-2 therapy (6, 17–19). Moreover, our finding that postoperative deterioration of glycemic control (Group 3) offsets favorable baseline status extends earlier observations by highlighting the prognostic salience of early post-PCI glycemic stabilization, rather than baseline HbA1c alone.

Furthermore, the outcome of group 2 was close to that of the continuously well-controlled group, with smaller differences in the risk of MACE (20.00% vs. 11.76%) and the mean LVEF (55.51% vs. 56.83%), suggesting that active postoperative blood glucose control can partially reverse the adverse effects of baseline blood glucose. This finding supports the importance of early intervention, especially for patients with poor baseline blood glucose control. Intensive postoperative management may significantly improve prognosis. It is notable that the baseline status of patients in Group 3 was better, but the postoperative blood glucose fluctuation significantly increased the risk of MACE and cardiogenic mortality. At the same time, the improvement rate of cardiac function and the quality of life score were both lower than those in Group 1 and Group 2. This phenomenon suggests that postoperative blood glucose fluctuations may counteract the benefits of early treatment and further exacerbate myocardial injury. The analysis of the reasons lies in that, first of all, repeated hypoglycemia activates the sympathetic nerve, increasing myocardial oxygen consumption and the risk of arrhythmia; Secondly, high-dose insulin may promote the proliferation of vascular smooth muscle and lipid deposition (22). In contrast, SGLT-2 inhibitors can reduce intracellular calcium overload in cardiomyocytes by inhibiting sodium-hydrogen exchangers (NHE), and at the same time promote ketone body utilization to improve energy metabolism (23). While GLP-1 receptor agonists can inhibit myocardial fibrosis through the AMPK pathway (24). Therefore, for elderly AMI patients, early combination with new hypoglycemic drugs may be more conducive to the recovery of cardiac function. In the future, the feasibility of “sequential hypoglycemic drug therapy” after surgery can be further explored. While our grouping based on change in HbA1c between baseline and 3 months indirectly captures clinically meaningful worsening or improvement, formal GV metrics (e.g., standard deviation, coefficient of variation, variability independent of the mean) typically require higher-frequency sampling or continuous glucose monitoring (CGM). Evidence suggests that greater in-hospital or short-term GV after AMI is associated with higher rates of 30-day MACE and mortality, independent of diabetes status, underscoring the potential importance of GV beyond mean HbA1c. We did not compute CGM-derived or high-frequency GV indices because only quarterly measurements were available; prospective CGM-enabled cohorts are warranted to validate whether GV adds independent prognostic information to 3-month HbA1c status in elderly AMI patients. In older AMI populations, revascularization strategy continues to influence outcomes, as highlighted in contemporary analyses of complete vs. culprit-only PCI; additionally, emerging data link type 2 diabetes and coronary microvascular dysfunction to MACE risk after PCI. These patterns support aggressive cardiometabolic optimization after AMI, including durable glycemic control (25).

Given that HbA1c at 3 months post-PCI strongly discriminated 1-year outcomes in elderly AMI patients, we recommend routine HbA1c monitoring every 3 months during the first post-PCI year, coupled with titration of antihyperglycemic therapy to maintain HbA1c ≤ 7% where appropriate and safe. In patients with diabetes or high cardiometabolic risk, early consideration of SGLT-2 inhibitors—in addition to standard post-AMI therapy—may confer incremental benefits for heart-failure prevention and renal protection, while GLP-1 receptor agonists can be considered when weight reduction or additional MACE lowering is prioritized. These choices should be individualized, accounting for age-related frailty, renal function, and hypoglycemia risk, and implemented alongside structured education and adherence support.

## 5 Conclusion

To sum up, the dynamic changes of postoperative blood glucose control can significantly affect the recovery of cardiac function and quality of life of elderly AMI patients. Optimizing the selection of hypoglycemic drugs (such as giving priority to the use of SGLT-2 inhibitors) and strengthening postoperative blood glucose management are the key strategies for improving prognosis. This study still has certain limitations: Firstly, this study is a single-center and observational study with a small sample size, and there may be selection bias. Furthermore, the dynamic changes of postoperative blood glucose control may be affected by multiple factors (such as medication compliance, lifestyle intervention, etc.), and these factors were not analyzed in detail in this study. Future studies can expand the sample size, adopt a multicenter design, and combine the specific mechanism of action of hypoglycemic drugs to further explore the long-term impact of postoperative blood glucose management on cardiac function recovery and quality of life. Furthermore, for high-risk patients with blood glucose fluctuations, intervention measures should be strengthened to reduce the risk of MACE and improve the quality of life.

## Data Availability

The raw data supporting the conclusions of this article will be made available by the authors, without undue reservation.
